# VRE and antibiotic use in German ICUs—an ecological analysis of 15 years of surveillance data

**DOI:** 10.1093/jacamr/dlaf216

**Published:** 2025-12-02

**Authors:** Juliane Karras, Frank Schwab, Friederike Maechler

**Affiliations:** Institute of Hygiene and Environmental Medicine, Charité—Universitätsmedizin Berlin, Corporate Member of Freie Universität Berlin and Humboldt-Universität zu Berlin, Campus Benjamin Franklin, Hindenburgdamm 27, Berlin 12203, Germany; Institute of Hygiene and Environmental Medicine, Charité—Universitätsmedizin Berlin, Corporate Member of Freie Universität Berlin and Humboldt-Universität zu Berlin, Campus Benjamin Franklin, Hindenburgdamm 27, Berlin 12203, Germany; National Reference Center for the Surveillance of Nosocomial Infections, Charité—Universitätsmedizin Berlin, Berlin, Germany; Institute of Hygiene and Environmental Medicine, Charité—Universitätsmedizin Berlin, Corporate Member of Freie Universität Berlin and Humboldt-Universität zu Berlin, Campus Benjamin Franklin, Hindenburgdamm 27, Berlin 12203, Germany; National Reference Center for the Surveillance of Nosocomial Infections, Charité—Universitätsmedizin Berlin, Berlin, Germany

## Abstract

**Background:**

This 15 years study reveals highlighting key differences in resistance trends and incidence of vancomycin-resistant *Enterococcus faecium* versus *Enterococcus faecalis* in German intensive care units (ICUs). By linking these patterns to antibiotic use, it uncovers crucial insights into the evolving battle against vancomycin-resistant enterococci (VRE) in critical care.

**Methods:**

A retrospective ecological cohort study using data from the German SARI (Surveillance of Antimicrobial Use and Antimicrobial Resistance in German ICUs) system was conducted from January 2006 to December 2020. Data from 79 ICUs were analysed. Incidence densities (ID) and resistance rates (RR) for *E*. *faecium* and *E*. *faecalis* were calculated, alongside antibiotic use densities in defined daily doses per 100 patient days. Generalized linear models and generalized estimating equations assessed temporal trends and associations with antibiotic consumption.

**Results:**

A total of 42 701 Enterococcus isolates were analysed: 21 672 *E*. *faecium* and 21 029 *E*. *faecalis*. VRE was found in 17.0% of *E*. *faecium* and 0.2% of *E. faecalis.* VR*E*. *faecium* showed a significant increase in ID and RR, while vancomycin-sensitive *E*. *faecium* decreased. VR*E*. *faecalis* remained rare. Antibiotic use patterns showed a significant increase in carbapenem (184.9%), glycopeptides (131.7%), and vancomycin (93.9%).

**Conclusions:**

This study highlights a sustained increase in the incidence and resistance of VR*E*. *faecium*. While glycopeptides are well-known contributors, carbapenem use may also play a role in VRE colonization, potentially through disruption of the microbiome. Further research is needed to clarify the complex relationship between antibiotic exposure, microbiome-related mechanisms, and resistance development.

## Introduction

Antimicrobial resistance (AMR) has emerged as one of the most significant global health threats, compromising the treatment of common infectious diseases and posing an escalating risk to public health worldwide. Among the most concerning pathogens exhibiting multidrug resistance are vancomycin-resistant enterococci (VRE) including *Enterococcus faecium* (*E. faecium*) and *Enterococcus faecalis* (*E. faecalis*), which have increasingly become a major cause of nosocomial infections, particularly in vulnerable populations such as those in intensive care units (ICUs).^1,2^

VRE, especially *E. faecium*, have acquired resistance to vancomycin and other classes of antibiotics, rendering them difficult to treat and associated with increased morbidity and mortality. The emergence and spread of VRE are driven by factors such as the overuse of antibiotics in healthcare settings, selective pressure resulting from inappropriate antimicrobial therapies, and the horizontal transmission of resistant strains within healthcare environments. The global dissemination of VRE has raised significant public health concerns, prompting the World Health Organization (WHO) to classify these pathogens as a critical priority for research and intervention.^3,4^

In Europe and other regions, surveillance data from organizations such as the European Centre for Disease Prevention and Control (ECDC) and national surveillance programmes emphasize a concerning rise in VRE prevalence, particularly in countries with high rates of antibiotic consumption.^5^ The European Surveillance System (TESSy) tracks the rise in VR*E. faecium* and VR*E. faecalis* across Europe, with Poland, Hungary, Germany and Italy showing the highest increases in resistance.^6^

In Germany, resistance rates (RRs) for *E. faecium* in blood cultures have risen steadily, from 17.4% in 2017 to 25.1% in 2019, with regional variation across the country.^7^ Data from the Robert Koch Institute (RKI) and the Paul Ehrlich Society further highlight this increasing trend in VRE infections.^8–10^ However, a comprehensive epidemiological understanding of invasive VRE in Europe and Germany remains lacking, and continuous monitoring of resistance patterns and antibiotic consumption is essential for controlling the spread of this threat.^7,8^

The study aims to investigate the trends in the development of RRs and densities of VR*E. faecium* and VR*E. faecalis* over a period of 15 years, with emphasis on the relationship between these trends and antibiotic consumption. Specifically, the study seeks to determine whether there were differences in the development of RRs between these two pathogens, and whether a shift from sensitive to resistant pathogens occurred while the overall pathogen load remained constant. Additionally, the study explores whether similar trends are observed in the application densities of antibiotics used to treat VRE infections, and how these changes in antibiotic use correlate with resistance trends, within the context of German ICUs.

## Methods

### Study design and data collection

We performed a retrospective ecological cohort study with a pre-determined time period of 15 years, starting from January 2006, including data up to December 2020.

Data were collected through the German surveillance system SARI (Surveillance of Antimicrobial Use and Antimicrobial Resistance in German Intensive Care Units), which gathers prospective data from ICUs and laboratories on 13 key pathogens responsible for nosocomial infections and related antibiotic treatments. Pathogen isolates were non-duplicates and were not differentiated by infection or colonization status.

All units participated voluntarily but needed to fulfil different requirements, e.g. implementation of external and internal quality processes, available laboratories that are able to determine antibiotic susceptibility following standardized procedures and guidelines (DIN 58940, NCCLS, CLSI or EUCAST).^11^

For the analyses, matched data were included from all units that delivered separate information regarding *E*. *faecium* and *E. faecalis* and the use of antibiotics. Relevant antimicrobial substances were analysed in sub-groups based on their Anatomical Therapeutic Chemical (ATC) classification.^12^ Numerator and denominator data per ICU and month were derived from separate databases. If either information was missing, the respective ward was excluded from the data set.

The SARI database is part of the German SIR (Spread of Nosocomial Infections and Resistant Pathogens) research network and was supported by the German Federal Ministry of Education and Research. All data used in this study were anonymized and collected in accordance with national guidelines for Good Epidemiological Practice (GEP),^13^ ensuring compliance with data protection and research ethics standards. In addition, the legal framework governing infection surveillance in Germany is defined by the Protection against Infection Act (*Infektionsschutzgesetz*, § 23),^14^ which requires all hospitals to continuously collect and analyse data on nosocomial infections and antimicrobial resistance.

### Statistical analyses

The incidence density (ID) of a pathogen was calculated as number of pathogens divided patient days and multiplied with 1000. The RR of pathogens was calculated from the number of resistant isolates per 100 tested pathogens.

Antibiotic consumption was expressed as antibiotic use density and based on the number of administered antibiotics in defined daily doses (DDD), (and) standardized per 100 patient days. One DDD represents the standard average treatment dose of an antimicrobial agent per day, as defined by the WHO.^15^

In the descriptive analyses, annual RRs, incidence densities, and antibiotic use densities were calculated. Crude rate ratios with corresponding 95% confidence intervals (CI) were determined to compare different time periods. To examine temporal development of pathogen occurrence and antibiotic use, both univariable and multivariable regression analyses were performed based on monthly ICU-aggregated data.

Generalized linear models (GLM) were used to estimate the association between the frequency of pathogens and the use of different antimicrobial groups in the current and previous month. Additional potential confounders such as trend (month/year), season, ICU type and size and hospital type, were incorporated into the models to control for temporal and structural variability that could influence the associations.

For the analysis of RRs, GLMs with a binomial distribution and logit link function were used. To examine incidence densities, GLMs with a negative binomial distribution were applied due to over dispersion. The Lagrange Multiplier test was used to assess whether the negative binomial model provided a significantly better fit than the Poisson model. The log of monthly patient days was used as an offset in the model.

For the analysis of antibiotic use densities, GLMs with a normal distribution were applied. To account for clustering effects within ICUs, adjusted incidence rate ratios (IRR) with 95% CI were estimated using generalized estimating equations (GEE) models with an autoregressive correlation structure.

To account for potential confounding factors and to identify independent associations between antibiotic use and VRE resistance trends, multivariate analyses were performed. Thus, in multivariable modelling, antibiotic use parameters of all antibiotic groups were considered in the analysis. All antibiotic parameters with a *P* value < 0.2 in a univariable model were included in the full model, and non-significant parameters were then removed stepwise. Model optimization was guided by the lowest χ^2^ value and Type III score statistic *P* value ≥ 0.05. The GEE model fit (or goodness-of-fit) was evaluated using the quasi-likelihood information criterion (QIC).

Antimicrobial agents specifically relevant for the treatment of infections with VRE, including daptomycin, linezolid (included in the group J01XX), and tigecycline (included in the group J01AA), were excluded from the analysis. Antibiotic use densities were standardized per 100 patient days, allowing model estimates to be interpreted as effects per one DDD per 100 patient days.

In the sensitivity analyses, final models were adjusted for time, ICU type, and hospital type to confirm the robustness of results. Models were also expanded to examine/explore potential lagged effects by analysing antibiotic use from the previous/preceding 2 months. To adjust for the selection pressure of relevant agents for the treatment of VRE, a further model, including these antibiotic groups, was calculated.

Collinearity of antibiotic use between the different antibiotic groups was investigated by calculating Spearman correlation coefficients. *P* values < 0.05 were considered statistically significant.

All analyses were performed using SPSS [IBM SPSS Statistics] and SAS 9.4 [SAS Institute].

## Results

Data from January 2006 to December 2020 were included in this study, comprising a total of 79 ICUs (see Table 1). The ICUs contributed data with a median of 34 038 patient days per unit and participated for a median duration of 103 months, interquartile range (IQR) 48–144, out of a possible 180 months over the 15-year study period.

**Table 1.** Structure of participating ICUs (*n* = 79) from 2006 until 2020

Parameter	Category	*N*
Type of ICU	Interdisciplinary	35 (44.3%)
	Medical	19 (24.1%)
	Surgical	25 (31.6%)
Size of ICU (beds)	<12 beds	33 (41.8%)
	≥12 beds	46 (58.2%)
Type of hospital	Uni/Maxi/SP	62 (78.5%)
	Other than above	17 (21.5%)
Size of hospital (beds)	<600 beds	27 (34.2%)
	≤600 beds	52 (65.8%)

ICU, intensive care unit; Uni, university hospital = affiliated with a university, providing patient care, research, and teaching; Maxi, maximum care hospital = offers the widest range of complex medical services^16^; SP, specialty hospital = focuses on specific medical specialties or treatment areas.

### Antibiotic resistance

In the period from January 2006 through December 2020, a total of 42 701 *Enterococci* isolates were tested, 21 672 *E*. *faecium* and 21 029 *E*. *faecalis*. Among these three, 3692 (17.0%) *E*. *faecium* and 48 *E*. *faecalis* (0.2%) were identified as VRE. The analysis revealed significant trends in the incidence densities and RRs of VR*E. faecium* and VR*E. faecalis* between 2006 and 2020 (see Table 2). All calculated results are available in the comprehensive table in the appendix (see Appendix, Table S1 (available as Supplementary data at *JAC-AMR* Online)).

**Table 2.** Development of *Enterococcus faecium* and *faecalis* isolates and their resistance to vancomycin in 79 ICUs participating in SARI project between 2006 and 2020, Germany

Parameter	Total years 2006–20	Year 2006	Year 2020	CRR 2020 versus 2006 (95% CI), *P* value	ARR 2020 versus 2006^a^ (95% CI), *P* value	ALT per month^b^ (95% CI), *P* value
No. ICU	79	44	38			
Observed months	7774	506	431			
No. Patient days	3 073 571	190 473	183 233			
*E. faecium*						
No. *E*. *faecium*	21 672	1198	1308			
VS*E*. *faecium*	17 980	1162	830			
ID VS*E*. *faecium*	5.85	6.10	4.53	0.74 (0.68–0.81), *P* < 0.001	0.84 (0.62–1.12), *P* = 0.236	0.999 (0.997–1.001), *P* = 0.200
No. VR*E*. *faecium*	3692	36	478			
ID VR*E*. *faecium*	1.20	0.19	2.61	13.8 (9.84–19.37), *P* < 0.001	15.36 (7.58–31.09), *P* < 0.001	1.016 (1.014–1.018), *P* < 0.001
RR VR*E*. *faecium*	17.04	3.01	36.54	12.16 (8.67–17.06), *P* < 0.001	16.83 (8.30–34.27), *P* < 0.001	1.017 (1.015–1.020), *P* < 0.001
*E. faecalis*						
No. *E. faecalis*	21 029	1576	997			
No. VS*E. faecalis*	20 981	1576	996			
ID VS*E*. *faecalis*	6.83	8.27	5.44	0.66 (0.61–0.71), *P* < 0.001	0.71 (0.53–0.95), *P* = 0.022	0.999 (0.998–1.001), *P* = 0.334
No. VR*E*. *faecalis*	48	0	1			
ID VR*E*. *faecalis*	0.02	0.00	0.01	ND, *P* = 0.490	ND	1.003 (0.998–1.008), *P* = 0.275
RR VR*E*. *faecalis*	0.23	0.00	0.10	ND, *P* = 0.388	ND	1.003 (0.998–1.008), *P* = 0.234

No., number; ICU, intensive care unit; VSE, vancomycin-sensible enterococci; VRE, vancomycin-resistant enterococci; ID, incidence density (per 1000 patient days); RR, resistant rate (per 100 pathogens); CRR, crude rate ratio; ARR, adjusted rate ratio; CI, confidence interval; ND, not defined.

^a^ARR 2020 versus 2006, compares year 2020 with year 2006 and were calculated by GEE model with monthly ICU-based data considering the cluster effect of the ICU.

^b^ALT, Adjusted linear trend per month, were calculated by GEE model with monthly ICU-based data considering the cluster effect of the ICU.

For *E. faecium*, the ID of vancomycin-sensitive strains significantly decreased over the whole study time. However, no significant change was observed in the adjusted rate ratio between 2006 and 2020, and the linear trend analysis did not indicate a significant temporal change. In comparison, the ID of VR*E. faecium* increased significantly from 2006 onward. The monthly linear trend for VR*E. faecium* also demonstrated a significant upward trajectory.

The RR for VR*E. faecium* showed a significant increase from 2006 to 2020. The adjusted RR was similarly high, and the linear trend confirmed a significant increase in resistance, further highlighting the growing burden of vancomycin resistance.

For *E. faecalis*, the ID of vancomycin-sensitive strains decreased over time, with a significant reduction observed in the crude rate ratio. Conversely, the ID of VR*E. faecalis* remained very low, with only one isolate recorded in 2020.

### Antibiotic use

Overall, the analysis of antibiotic consumption reflects a broad range in the trend of different substances, including results for selected antibiotic substance classes (see Appendix, Table S2).

Although the use of antibiotics such as beta-lactamase inhibitors and fluoroquinolones declined markedly, the consumption of other antimicrobial classes increased over time. The trends for most antibiotics were statistically significant, indicating notable shifts in prescribing patterns over the 15-year period. Especially the consumption of carbapenems and combination penicillin increased over time.

The consumption of the broad-spectrum antibiotics (see J01CA, J01CR, J01DD, J01DE, J01DH, J01MA in the Appendix, Table S2) increased substantially, with carbapenem use rising by 184.9%. This upward trend is statistically significant for both monthly and annual consumption (see Table 3).

**Table 3.** Yearly development of antibiotic use densities for selected antibiotic groups and substances in 79 ICUs participating in SARI project between 2006 and 2020, Germany

Parameter	Year 2006	Year 2020	Increase 2020 versus 2006	% Increase 2020 versus 2006	ALT per month^a^ (95% CI), *P* value	ALT per year^a^ (95% CI), *P* value
No. ICU	44	38				
No. patient days	190 473	183 233				
Antibiotic use density (DDD/100 patient days) (ATC-code)						
Beta-lactamase sensitive penicillines (J01CE)	2.3	2.5	0.3	11.7	0.004 (−0.001–0.008), *P* = 0.059	0.0462 (−0.0022–0.0945), *P* = 0.061
Beta-lactamase resistant penicillines (J01CF)	3.6	9.5	5.9	165.2	0.050 (0.035–0.064), *P* < 0.001	0.5869 (0.4149–0.759), *P* < 0.001
Carbapenems (J01DH)	12.0	34.2	22.2	184.9	0.108 (0.068–0.148), *P* < 0.001	1.1707 (0.7107–1.6306), *P* < 0.001
Glycopeptides (J01XA)	4.0	9.3	5.3	131.7	0.026 (0.007–0.0441), *P* = 0.007	0.2745 (0.0601–0.4889), *P* = 0.012
Vancomycin (J01XA01) (p)	3.8	7.3	3.5	93.9	0.016 (0.002–0.030), *P* = 0.027	0.1634 (0.0013–0.3256), *P* = 0.048
Vancomycin (J01XA01) (o)	<0.01	<0.01	<0.01	−4.9	0.000 (−0.0001–0.0002), *P* = 0.647	0.0005 (−0.0017–0.0027), *P* = 0.643
Teicoplanin (J01XA02) (p)	0.2	2.0	1.8	766.6	0.009 (−0.002–0.022), *P* = 0.117	0.1096 (−0.0277–0.2468), *P* = 0.118
Aminoglycosides (J01G)	3.0	4.0	1.1	36.0	0.006 (−0.004–0.015), *P* = 0.221	0.0656 (−0.0449–0.176), *P* = 0.245

No., number; ICU, intensive care unit; ATC-code, anatomical therapeutic chemical (ATC) code; DDD, defined daily doses; p, parenteral; o, oral; ALT, adjusted linear trend; CI, confidence interval.

^a^ALT per month/year, were calculated by GEE model with ICU-based data considering the cluster effect of the ICU.

The consumption of antibiotics of the group of glycopeptides, including vancomycin and teicoplanin, also increased over the study period, with a significant increase in consumption per month and per year. The total use of glycopeptides rose by 131.7%, with vancomycin alone showing an increase of 93.9% (see Table 3).

Next, the consumption of aminoglycosides increased by 36% between 2006 and 2020. The observed monthly and annual trends (0.0058 DDD per 100 patient days per month and 0.0656 DDD per 100 patient days per year) were not statistically significant.

The analysis of collinearity between different antibiotic groups using Spearman correlation coefficients revealed only weak associations (see Appendix, Table S5). The highest observed correlations were between penicillin use and the time trend (ρ = 0.44), as well as between the use of carbapenems and glycopeptides (ρ = 0.44). None of the correlation coefficients exceeded 0.5, indicating that there were no strong correlations between the variables. Thus, these results suggest low collinearity.

### Antibiotic resistance in relation to antimicrobial use

The multivariable analyses for the occurrence of VS*E*. *faecium* and *faecalis*, and VR*E*. *faecium* and *faecalis* showed different results with respect to ID (Table 4) and RR (Table 5).

**Table 4.** Multivariate analyses for the outcome incidence density (ID) of vancomycin-resistant *Enterococcus faecium* and *faecalis*

		VR*E*. *faecium*		VR*E*. *faecalis*	
Parameter	Category	IRR (95% CI)	*P* value	IRR (95% CI)	*P* value
Parsimonious model (with time)					
Time trend (linear)	Per month	1.015 (1.013–1.017)	<0.001	1.003 (0.998–1.008)	0.216
Carbapeneme (J01DH)	Per 1DDD/100 pd	1.009 (1.006–1.012)	<0.001	—	—
Glycopeptide (J01XA)	Per 1DDD/100 pd	1.008 (1.002–1.014)	0.009	—	—
Imidazole derivatives (J01XD)	Per 1DDD/100 pd	1.027 (1.011–1.043)	0.001	—	—
Beta-lactamase sensitive penicillin (J01CE)	Per 1DDD/100 pd	—	—	0.88 (0.81–0.956)	0.003
Model adjusted by confounders (time/type ICU/hospital)					
Time trend (linear)	Per month	1.015 (1.013–1.018)	<0.0001	1.003 (0.997–1.008)	0.331
Type of ICU	Medical	0.978 (0.682–1.4)	0.902	0.831 (0.297–2.329)	0.725
	Surgical	1.053 (0.698–1.587)	0.807	1.215 (0.479–3.08)	0.682
	Interdisciplinary	1 = reference		1 = reference	
Type of hospital	Maximum care	1.446 (0.847–2.469)	0.177	0.508 (0.128–2.018)	0.336
	Other	1 = reference		1 = reference	
Carbapeneme (J01DH)	Per 1DDD/100 pd	1.008 (1.005–1.011)	<0.001	—	—
Glycopeptide (J01XA)	Per 1DDD/100 pd	1.006 (1–1.013)	0.046	—	—
Imidazole derivatives (J01XD)	Per 1DDD/100 pd	1.026 (1.011–1.042)	0.001	—	—
Beta-lactamase sensitive penicillin (J01CE)	Per 1DDD/100 pd	—	—	0.888 (0.822–0.96)	0.003
Parsimonious model with time lag of AB use (without tetracyclines^a^/Other antibacterials^b^)					
Time trend (linear)	Per month	1.015 (1.012–1.017)	<0.001	—	—
Carbapeneme (J01DH) in the current month	Per 1DDD/100 pd	1.008 (1.005–1.011)	<0.001	—	—
Glycopeptide (J01XA) in the current month	Per 1DDD/100 pd	1.006 (1–1.012)	0.033	—	—
Imidazole derivatives (J01XD) in the current month	Per 1DDD/100 pd	1.027 (1.012–1.044)	0.001	—	—
Carbapeneme (J01DH) 1 month before the current month	Per 1DDD/100 pd	1.006 (1.002–1.009)	0.002	—	—
Aminoglycosides (J01G) 2 month before the current month	Per 1DDD/100 pd	1.008 (1.003–1.012)	0.001	—	—
Beta-lactamase sensitive penicillin (J01CE)	Per 1DDD/100 pd	—	—	0.883 (0.812–0.959)	0.003

VRE, vancomycin-resistant enterococcus; RD, resistance density; IRR, incidence rate ratio; CI, confidence interval; pd, patient days.

^a^Tigeicyclin (J01AA12) (69% of use in the group J01AA).

^b^Linezolid (J01XX08) & Daptomycin (J01XX09) (79% of use in the group J01XX in the year 2020).

**Table 5.** Multivariate analyses for the outcome resistance rate of vancomycin-resistant *Enterococcus faecium* and *faecalis*

Parameter	Category	VR*E*. *faecium*		VR*E*. *faecalis*	
		IRR (95% CI)	*P* value	IRR (95% CI)	*P* value
Parsimonious model (with time)					
Time trend (linear)	Per month	1.017 (1.015–1.019)	<0.001	1.003 (0.998–1.009)	0.218
Glycopeptide (J01XA)	Per 1DDD/100 pd	1.015 (1.004–1.028)	0.011	—	—
Aminoglycosides (J01G)	Per 1DDD/100 pd	1.014 (1.004–1.024)	0.005	—	—
Beta-lactamase sensitive penicillin (J01CE)	Per 1DDD/100 pd	—	—	0.916 (0.869–0.964)	0.001
Model adjusted by confounders (time/Type ICU/hospital)					
Time trend (linear)	Per month	1.017 (1.015–1.019)	<0.001	1.003 (0.998–1.009)	0.267
Type of ICU	Medical	1.295 (0.73–2.299)	0.377	1.107 (0.417–2.939)	0.838
	Surgical	1.079 (0.602–1.936)	0.798	1.267 (0.513–3.132)	0.608
	Interdisciplinary	1 = reference		1 = reference	
Type of hospital	Maximum care	1.178 (0.588–2.358)	0.644	0.449 (0.143–1.414)	0.171
	Other	1 = reference		1 = reference	
Glycopeptide (J01XA)	Per 1DDD/100 pd	1.014 (1.003–1.026)	0.016	—	—
Aminoglycosides (J01G)	Per 1DDD/100 pd	1.014 (1.004–1.023)	0.005	—	—
Beta-lactamase sensitive penicillin (J01CE)	Per 1DDD/100 pd	—	—	0.917 (0.874–0.961)	<0.001
Parsimonious model with time lag of AB use (without tetracyclines^a^/Other antibacterials^b^)					
Time trend (linear)	Per month	1.017 (1.014–1.019)	<0.001	—	—
Glycopeptide (J01XA) in the current month	Per 1DDD/100 pd	1.011 (1.003–1.018)	0.007	—	—
Aminoglycosides (J01G) in the current month	Per 1DDD/100 pd	1.009 (1.001–1.018)	0.031	—	—
Penicillins with extended spectrum (J01CA) 1 month before the current month	Per 1DDD/100 pd	1.005 (1–1.01)	0.036	—	—
Glycopeptide (J01XA) 1 month before the current month	Per 1DDD/100 pd	1.014 (1.007–1.021)	<0.001	—	—
Glycopeptide (J01XA) 2 months before the current month	Per 1DDD/100 pd	1.007 (1.002–1.013)	0.013	—	—
Aminoglycosides (J01G) 2 months before the current month	Per 1DDD/100 pd	1.01 (1.002–1.017)	0.011	—	—
Beta-lactamase sensitive penicillin (J01CE)	Per 1DDD/100 pd	—	—	0.916 (0.869–0.964)	0.001

VRE, vancomycin-resistant enterococcus; RR, resistance rate; IRR, incidence rate ratio; CI, confidence interval; pd, patient days.

^a^Tigeicyclin (J01AA12) (69% of use in the group J01AA).

^b^Linezolid (J01XX08) & Daptomycin (J01XX09) (79% of use in the group J01XX in the year 2020).

## Discussion

The study found that, since 2006, the ID of VR*E. faecium* increased steadily by 1.5% per year (see Table 4), with an annual rise in the RR of 1.7% (see Table 5). Although VS*E. faecium* strains remained stable, *E. faecium* strains showed overall higher proportions in vancomycin resistance. These findings align with previous studies and recent data from the European Antimicrobial Resistance Surveillance (EARS) network.^17^ However, the upward trend of VR*E*. *faecium* resistance remains concerning and reflects the broader situation in Germany, where increasing RRs were observed across various hospital types and ICUs.^9^ A recent report also identified VR*E*. *faecium* as the primary pathogen in all evaluated outbreak events in Germany and the Netherlands.^18^

From 2006 to 2019, the ID of VR*E*. *faecium* rose by a factor of 17. However, in 2020, a trend reversal was noted in Germany, while other European countries continued to report increasing RRs.^19^ The notable shift in data between 2019 and 2020 may be attributable to the COVID-19 pandemic, which led to a reorganization of ICU resources, a reduction in surgical procedures, and changes in patient profiles, factors that are not captured by the SARI system. The German RKI also reported a decline in pathogen detection during the pandemic, suggesting a possible decrease in data recording or pathogen screening following the KRINKO recommendations published in 2018 on infection prevention and control in ICUs.^9,20,21^

Beyond these structural and operational influences, the observed fluctuations in VRE rates may also reflect underlying microbiological dynamics. Notably, recent genomic surveillance in German hospitals has documented the rise and replacement of dominant clonal lineages of multidrug-resistant *Enterococcus faecium*, including shifts in prevalent sequence types over time, such as the emergence and expansion of the ST117/CT71 lineage.^8,22^ Such clonal turnover can substantially affect aggregated resistance data, as the expansion of a particularly fit or transmissible clone may temporarily elevate RRs independent of antibiotic use, while its decline can lead to apparent reductions even under constant selective pressure.^22^

Published studies have reported high RRs of VR*E*. *faecium* associated with high application densities of glycopeptides, aminoglycosides, and carbapenems.^23,24^ Our VR*E*. *faecium* ID data underscore the contribution of these three to the rise in VRE, with carbapenem use showing a strong association both concurrently and in the preceding months.

Although carbapenems do not directly target enterococci, their use has been shown to contribute to the spread of VRE.^24^ Moreover, pioneering work from Brandl *et al*. revealed that lipopolysaccharide and flagellin of Gram-negative pathogens stimulate the production of a C-type lectin, called REGIIIγ, via an interaction with toll-like receptors. This C-type lectin exhibits antimicrobial activity against Gram-positive bacteria.^25^ The depletion of Gram-negative microbiota through antibiotic use also suppresses REGIIIγ production, which normally helps to control Gram-positive bacteria, thereby creating an environment that favours the overgrowth of VRE in the gastrointestinal tract. Thus, antimicrobials such as carbapenems that are mainly used in Gram-negative infections, may also promote VRE colonization in the intestinal microbiome.^24,26,27^ The correlation between VR*E*. *faecium* and the use of carbapenems is also supported by earlier study findings from Taiwan, where data were evaluated from 2010 to 2019, examining the prevalence of VR*E*. *faecium* and antibiotic consumption. In this study, increased consumption of carbapenems, which included meropenem, was also found to be significant for the resistance development of VR*E*. *faecium*. Although the study explored data from only one hospital, the results clearly indicate that significant changes in antimicrobial use, e.g. of carbapenems, affected antimicrobial resistance of enterococci in that hospital.^28^

In addition, the route of antibiotic administration is a crucial factor influencing resistance development. A study has shown that oral vancomycin, commonly used for treating, i.e. Clostridioides difficile infections, achieves high concentrations within the gut lumen, significantly disrupting the intestinal microbiota and promoting selective overgrowth and persistence of vancomycin-resistant enterococci. In contrast, intravenous vancomycin has limited direct impact on the gut flora, making it less likely to contribute to intestinal VRE colonization.^29^ However, within the scope of our available data, no definitive conclusions can be drawn regarding the impact of oral versus intravenous vancomycin on VRE colonization, as cases involving oral administration were limited, with the majority of vancomycin use recorded as parenteral.

In our study, the group of carbapenems did affect the ID but not the RR of VR*E*. *faecium*. This counterintuitive observation may be explained by the fact that RRs are biased towards patients and periods of time with the pathogens of interest and thus represent a specific, more vulnerable patient population, i.e. patients already colonized with *E. faecium*. The exposure of hospitalized patients to antibiotics with activity against Gram-negative bacteria and Gram-positive species, excluding *E*. *faecium*, results in substantial changes in the gut microbiota and overgrowth with *E. faecium*, both VRE and VSE.^24^ Consequently, the potential role of carbapenems as primary contributors to the emergence of VRE may be of reduced significance when considering patients already colonized with *E. faecium.*

In addition, RRs can disproportionately reflect specific periods of time (e.g. periods with enhanced surveillance screening activity such as outbreaks), as months without identified pathogens are excluded from the data. In contrast, incidence densities provide a more comprehensive view, capturing all patients regardless of whether they are carriers of the respective pathogen.

The study has several limitations. First, the data are aggregated at the ward level rather than the individual patient level, so it is not possible to link specific antibiotics or pathogens to individual patients, and the ecological study design does not allow for establishing causal relationships.

Second, there were variations between participating ICUs, as not all units provided continuous data for the full 15-year period, impacting data reliability. Greater participation and consistent data collection would have improved the results, but this is difficult to achieve on a voluntary basis.

Third, the overrepresentation of university and maximum-care hospitals may lead to an overestimation of antibiotic use and RRs. While a correlation may exist at lower care levels, it is likely masked/overshadowed by the high case numbers from larger hospitals.

Fourth, variations in resistance testing standards and procedures across laboratories need to be taken into account, as they could affect the results, especially over time, as testing methods gradually evolve.^30^ The breakpoints for antimicrobial susceptibility testing differ between EUCAST (European Committee on Antimicrobial Susceptibility Testing) and CLSI (Clinical and Laboratory Standards Institute), resulting in inconsistent interpretations of resistance profiles. For example, the breakpoint for vancomycin resistance in *E. faecium* is set at 32 µg/mL by EUCAST and 64 µg/mL by CLSI.^31,32^ Such discrepancies may affect the reporting outcomes, as the susceptibility and resistance classifications of an organism depend on the standard used.^31,33^ Over the study period, EUCAST adoption increased slightly, with ICUs using DIN/EUCAST rising from about 55% in 2006 to 64% in 2019. However, these gradual methodological shifts are unlikely to explain the observed rise in VRE rates.

Fifth, we were unable to distinguish between hospital- and community-acquired infections, as data on the onset of VSE and VRE colonization were not available. In this context, it is critical to consider that antibiotic use in non-ICU settings has been shown to contribute to the overall prevalence of VRE in the hospital setting, with effects that may manifest weeks to months after exposure and are therefore not captured in our analyses.^34^ Moreover, pathogen colonization, including VRE, is often already present at ICU admission due to ambulant or non-ICU related antibiotic therapy, underscoring the impact of prior exposures outside the ICU environment.^35^ This limitation hinders the assessment of key risk factors such as prolonged hospital stays, previous medication, or the use of medical devices, which contribute to VRE colonization and infection.^36^

Sixth, the statistical analysis of the less frequently observed VR*E*. *faecalis* requires further discussion. The study results indicate a stable occurrence of the resistant strains, with VS*E*. *faecalis* showing neither a significant increase nor a decrease over time. Although no specific antibiotic class was identified as a risk factor for RR or ID, the challenge of inestimable pathogen counts must be emphasized. Missing observations and resulting data gaps led to non-calculable outcomes, preventing a reliable interpretation. However, the literature consistently reports a lower prevalence of VR*E*. *faecalis* compared with VR*E*. *faecium*,^33,36,37^ which is in line with the findings of this retrospective study.

Lastly, the 2018 update of the German IPC guidelines on the management of resistant enterococci may have reduced the number of VRE screening tests. The previous broad screening strategy, especially for high-risk patients, was replaced with a more targeted approach to optimize resource allocation and improve efficiency.^38^ While this may have led to fewer VRE screenings, the trend is not evident in recent national ICU surveillance data.^39,40^ According to unpublished KISS surveillance data,^41^ the overall proportion of ICUs performing VRE screening has remained relatively stable from 2006 through 2020, indicating that the policy change from 2018 has not yet had a measurable impact within our 15-year dataset. This is particularly relevant as the level of screening influences both ID and RRs; stable screening practices, therefore, support the validity of temporal trends observed in both indicators. Additionally, similar developments are observed in bloodstream infection data from EARS-Net, which are independent of admission screening and therefore strengthen the reliability of the observed VRE patterns, a trend perfectly mirrored by the expansion and contraction of the aforementioned ST117 CT71 strain.

### Conclusion

This study demonstrates a continuous increase in the incidence and resistance of VR*E. faecium* over time, particularly in hospital settings. Beyond the established role of glycopeptides, carbapenem use may also contribute to VRE colonization, likely through microbiome disruption. However, further research is needed to better understand the complex interplay between antibiotic exposure, microbiome-related molecular mechanisms, and the development of resistance. Strengthening the systematic linkage of antibiotic use and resistance data are essential to inform targeted antimicrobial stewardship and infection control strategies.

## Supplementary Material

dlaf216_Supplementary_Data

## Supplementary data

Tables S1–S5 and are available as Supplementary data at *JAC-AMR* Online.
