# Supplementary material for: VRE and antibiotic use in German ICUs—an ecological analysis of 15 years of surveillance data
Source: JAC Antimicrob Resist. 2025 Dec 2;7(6):dlaf216. doi: 10.1093/jacamr/dlaf216 (PMC12969279; doi:10.1093/jacamr/dlaf216)
Supplement: dlaf216_Supplementary_Data [file dlaf216_supplementary_data.docx]

**Appendix**

**Supplemental Table 1**: Yearly development of *Enterococcus* f*aecium* and *faecalis* isolates and their resistance to Vancomycin on 79 ICUs participating in SARI project between 2006 and 2020, Germany.

| **Parameter** | **total** | **2006** | **2007** | **2008** | **2009** | **2010** | **2011** | **2012** | **2013** | **2014** | **2015** | **2016** | **2017** | **2018** | **2019** | **2020** | **CRR 2020 vs. 2006 (95%C), p-value** | **ARR 2020 vs. 2006** (95%C), p-value** | **ALT per month *** (95%CI),**  **p-value** |
| --- | --- | --- | --- | --- | --- | --- | --- | --- | --- | --- | --- | --- | --- | --- | --- | --- | --- | --- | --- |
| No. ICU | 79 | 44 | 43 | 43 | 45 | 43 | 43 | 52 | 50 | 46 | 46 | 49 | 45 | 44 | 43 | 38 |  |  |  |
| Observed months | 7774 | 506 | 501 | 489 | 515 | 464 | 492 | 593 | 591 | 529 | 542 | 557 | 528 | 522 | 514 | 431 |  |  |  |
| No. Patient days | 3073571 | 190473 | 188178 | 176840 | 189999 | 182871 | 194699 | 232181 | 235490 | 201208 | 215637 | 228185 | 223281 | 220555 | 210741 | 183233 |  |  |  |
| *E. faecium* |  |  |  |  |  |  |  |  |  |  |  |  |  |  |  |  |  |  |  |
| No*. E. faecium* | 21672 | 1198 | 1264 | 1093 | 1178 | 1225 | 1421 | 1354 | 1613 | 1364 | 1503 | 1756 | 1804 | 1818 | 1773 | 1308 |  |  |  |
| VS*E. faecium* | 17980 | 1162 | 1220 | 1012 | 1098 | 1140 | 1260 | 1222 | 1398 | 1231 | 1272 | 1395 | 1405 | 1255 | 1080 | 830 |  |  |  |
| ID VS*E. faecium* | 5.85 | 6.10 | 6.48 | 5.72 | 5.78 | 6.23 | 6.47 | 5.26 | 5.94 | 6.12 | 5.90 | 6.11 | 6.29 | 5.69 | 5.12 | 4.53 | 0.74  (0.68-0.81) p<0.001 | 0.84  (0.62-1.12) p=0.236 | 0.999  (0.997-1.001) p=0.200 |
| No. VR*E. faecium* | 3692 | 36 | 44 | 81 | 80 | 85 | 161 | 132 | 215 | 133 | 231 | 361 | 399 | 563 | 693 | 478 |  |  |  |
| ID VR*E. faecium* | 1.20 | 0.19 | 0.23 | 0.46 | 0.42 | 0.46 | 0.83 | 0.57 | 0.91 | 0.66 | 1.07 | 1.58 | 1.79 | 2.55 | 3.29 | 2.61 | 13.8  (9.84-19.37)  p<0.001 | 15.36  (7.58-31.09)  p<0.001 | 1.016  (1.014-1.018) p<0.001 |
| RR VR*E. faecium* | 17.04 | 3.01 | 3.48 | 7.41 | 6.79 | 6.94 | 11.33 | 9.75 | 13.33 | 9.75 | 15.37 | 20.56 | 22.12 | 30.97 | 39.09 | 36.54 | 12.16  (8.67-17.06) p<0.001 | 16.83  (8.30-34.27) p<0.001 | 1.017  (1.015-1.020) p<0.001 |
| *E. faecalis* |  |  |  |  |  |  |  |  |  |  |  |  |  |  |  |  |  |  |  |
| No*. E. faecalis* | 21029 | 1576 | 1483 | 1283 | 1343 | 1219 | 1309 | 1511 | 1516 | 1209 | 1405 | 1542 | 1751 | 1480 | 1405 | 997 |  |  |  |
| No. VS*E. faecalis* | 20981 | 1576 | 1482 | 1280 | 1343 | 1215 | 1306 | 1506 | 1505 | 1201 | 1405 | 1541 | 1750 | 1473 | 1402 | 996 |  |  |  |
| ID VS*E. faecalis* | 6.83 | 8.27 | 7.88 | 7.24 | 7.07 | 6.64 | 6.71 | 6.49 | 6.39 | 5.97 | 6.52 | 6.75 | 7.84 | 6.68 | 6.65 | 5.44 | 0.66  (0.61-0.71)  p<0.001 | 0.71  (0.53-0.95) p=0.022 | 0.999  (0.998-1.001) p=0.334 |
| No. VR*E. faecalis* | 48 | 0 | 1 | 3 | 0 | 4 | 3 | 5 | 11 | 8 | 0 | 1 | 1 | 7 | 3 | 1 |  |  |  |
| ID VR*E. faecalis* | 0.02 | 0.00 | 0.01 | 0.02 | 0.00 | 0.02 | 0.02 | 0.02 | 0.05 | 0.04 | 0.00 | 0.00 | 0.00 | 0.03 | 0.01 | 0.01 | ND,  p=0.490 | ND | 1.003  (0.998-1.008)  p=0.275 |
| RR VR*E. faecalis* | 0.23 | 0.00 | 0.07 | 0.23 | 0.00 | 0.33 | 0.23 | 0.33 | 0.73 | 0.66 | 0.00 | 0.06 | 0.06 | 0.47 | 0.21 | 0.10 | ND,  p=0.388 | ND | 1.003  (0.998-1.008)  p=0.234 |

VSE, Vancomycin-sensible Enterococci; VRE, Vancomycin-resistant enterococci; ID, incidence density (per 1000 patient days); RR, resistant rate (per 100 pathogens); CRR, Crude Rate Ratio; ARR, Adjusted Rate Ratio; CI, confidence interval; ND, not defined; *ARR 2019 vs 2006, compares year 2019 with year 2006 and were calculated by GEE model with monthly ICU-based data considering the cluster effect of the ICU; **ARR 2020 vs 2006, compares year 2020 with year 2006 and were calculated by GEE model with monthly ICU-based data considering the cluster effect of the ICU; ***ALT per month, were calculated by GEE model with monthly ICU-based data considering the cluster effect of the ICU.

**Supplemental Table 2**: Yearly development of antibiotic use densities for selected antibiotic groups and substances on 79 ICUs participating in SARI project between 2006 and 2020, Germany [1/2].

| **Parameter** | **2006** | **2007** | **2008** | **2009** | **2010** | **2011** | **2012** | **2013** | **2014** | **2015** | **2016** | **2017** | **2018** | **2019** | **2020** | **Increase**  **2020 vs. 2006** | **% Increase**  **2020 vs. 2006** | **ALT per month**  **(95%CI)** | **p-value** | **ALT per year**  **(95%CI)** | **p-value** |
| --- | --- | --- | --- | --- | --- | --- | --- | --- | --- | --- | --- | --- | --- | --- | --- | --- | --- | --- | --- | --- | --- |
| No. ICU (N=79) | 44 | 43 | 43 | 45 | 43 | 43 | 52 | 50 | 46 | 46 | 49 | 45 | 44 | 43 | 38 |  |  |  |  |  |  |
| Antibiotic use density (DDD/100 patient days) (ATC-code) |  |  |  |  |  |  |  |  |  |  |  |  |  |  |  |  |  |  |  |  |  |
| ANTIBACTERIALS FOR SYSTEMIC USE (J01) | 138.0 | 138.0 | 130.0 | 143.1 | 140.5 | 144.7 | 146.3 | 143.0 | 138.9 | 151.6 | 150.1 | 148.0 | 150.9 | 152.6 | 158.9 | 20.9 | 15.1 | 0.112  (-0.013, 0.237) | 0.079 | 0.779  (-0.674, 2.232) | 0.293 |
| Beta-lactamase sensitive penicilline (J01CE) | 2.3 | 2.0 | 1.8 | 2.1 | 2.1 | 1.9 | 1.9 | 1.7 | 2.3 | 2.7 | 2.4 | 2.1 | 2.4 | 2.6 | 2.5 | 0.3 | 11.7 | 0.004  (-0.0001, 0.008) | 0.059 | 0.046  (-0.002, 0.095) | 0.061 |
| Penicillins with extended spectrum (J01CA) | 10.1 | 10.4 | 8.1 | 8.9 | 6.7 | 6.0 | 4.7 | 3.1 | 3.7 | 3.8 | 4.0 | 6.5 | 5.8 | 5.7 | 5.8 | -4.4 | -43.1 | -0.028  (-0.045, -0.009) | 0.002 | -0.339  (-0.552, -0.127) | 0.002 |
| Beta-lactamase resistant penicilline (J01CF) | 3.6 | 4.5 | 2.2 | 2.2 | 2.6 | 3.3 | 3.5 | 3.6 | 5.1 | 5.5 | 7.8 | 8.8 | 8.7 | 8.9 | 9.5 | 5.9 | 165.2 | 0.049  (0.035, 0.064) | <0.001 | 0.587  (0.415, 0.76) | <0.001 |
| Beta-lactamase  Inhibitor (J01CG) | 19.1 | 17.5 | 14.4 | 16.9 | 11.8 | 8.1 | 4.4 | 0.3 | 0.0 | 0.5 | 0.1 | 1.8 | 1.2 | 0.7 | 0.3 | -18.8 | -98.4 | -0.125  (-0.163, -0.088) | <0.001 | -1.47  (-1.914, -1.029) | <0.001 |
| Combinations of penicillins, incl. beta-lactamase inhibitors (J01CR) | 15.8 | 16.0 | 14.4 | 13.3 | 16.0 | 19.4 | 22.5 | 26.1 | 26.6 | 27.9 | 27.4 | 28.5 | 32.8 | 35.8 | 35.6 | 19.8 | 125.7 | 0.149  (0.119, 0.179) | <0.001 | 1.718  (1.360, 2.077) | <0.001 |
| First-generation cephalosporins (J01DB) | 3.9 | 3.3 | 2.3 | 2.9 | 2.9 | 3.5 | 3.9 | 3.9 | 3.9 | 3.5 | 3.2 | 3.5 | 4.1 | 4.2 | 3.7 | -0.2 | -5.2 | 0.009  (-0.008, 0.027) | 0.301 | 0.070  (-0.137, 0.277) | 0.507 |
| Second-generation cephalosporins (J01DC) | 9.4 | 9.8 | 10.0 | 9.6 | 9.1 | 9.2 | 8.6 | 8.7 | 7.4 | 9.4 | 7.2 | 6.2 | 4.6 | 4.0 | 3.3 | -6.1 | -64.6 | -0.042  (-0.059, -0.024) | <0.001 | -0.526  (-0.740, -0.312) | <0.001 |
| Third-generation cephalosporins (J01DD) | 12.3 | 11.2 | 10.6 | 11.9 | 11.9 | 11.7 | 10.9 | 10.1 | 9.1 | 9.9 | 9.1 | 9.9 | 8.2 | 8.1 | 7.6 | -4.7 | -38.3 | -0.029  (-0.049, -0.009) | 0.004 | -0.356  (-0.597, -0.115) | 0.004 |
| Fourth-generation cephalosporins (J01DE) | 1.1 | 1.0 | 0.4 | 0.4 | 0.4 | 0.4 | 1.5 | 1.4 | 0.5 | 0.6 | 1.1 | 1.7 | 1.7 | 1.7 | 2.2 | 1.1 | 99.8 | 0.007  (-0.001, 0.014) | 0.058 | 0.078  (-0.003, 0.160) | 0.059 |
| Carbapenemes (J01DH) | 12.0 | 13.3 | 15.0 | 17.9 | 18.7 | 21.7 | 23.5 | 24.5 | 25.2 | 25.2 | 27.4 | 24.6 | 27.9 | 30.7 | 34.2 | 22.2 | 184.9 | 0.108  (0.069, 0.148) | <0.001 | 1.171  (0.711, 1.631) | <0.001 |
| Glycopeptides (J01XA) | 4.0 | 3.8 | 3.7 | 4.6 | 6.2 | 5.6 | 5.7 | 6.3 | 6.3 | 5.8 | 6.7 | 5.9 | 6.5 | 7.8 | 9.3 | 5.3 | 131.7 | 0.026  (0.007, 0.044) | 0.006 | 0.275  (0.060, 0.489) | 0.012 |
| Vancomycin (p) (J01XA01) | 3.8 | 3.5 | 3.5 | 4.5 | 6.0 | 5.4 | 5.4 | 5.8 | 6.0 | 5.5 | 6.1 | 5.8 | 5.6 | 5.5 | 7.3 | 3.5 | 93.9 | 0.016  (0.002, 0.030) | 0.027 | 0.163  (0.001, 0.326) | 0.048 |
| Vancomycin (o)  (J01XA01) | <0.01 | <0.01 | <0.01 | <0.01 | <0.01 | <0.01 | <0.01 | <0.01 | <0.01 | <0.01 | <0.01 | <0.01 | <0.01 | <0.01 | <0.01 | <0.01 | -4.9 | 0  (-0.0001, 0.0002) | 0.647 | 0.0005  (-0.0017, 0.0027) | 0.643 |
| Teicoplanin (p)  (J01XA02) | 0.2 | 0.3 | 0.2 | 0.1 | 0.2 | 0.1 | 0.3 | 0.5 | 0.3 | 0.3 | 0.5 | 0.2 | 0.8 | 2.3 | 2.0 | 1.8 | 766.6 | 0.009  (-0.002, 0.022) | 0.117 | 0.110  (-0.028, 0.247) | 0.118 |
| Fluorochinolone  (J01MA) | 17.2 | 16.9 | 16.9 | 17.8 | 17.2 | 18.5 | 18.3 | 16.3 | 15.1 | 17.2 | 16.7 | 15.8 | 12.9 | 9.0 | 9.2 | -8.0 | -46.8 | -0.038  (-0.060, -0.017) | 0.001 | -0.495  (-0.752, -0.238) | 0.001 |

No., number; ICU, intensive care unit; ; ATC-code, Anatomical Therapeutic Chemical (ATC) code; DDD, defined daily doses; p, parenteral; o, oral; ALT, Adjusted Linear Trend; CI, confidence interval; ND, not defined; *ALT per month/year, were calculated by GEE model with ICU-based data considering the cluster effect of the ICU.

**Supplemental Table 2**: Yearly development of antibiotic use densities for selected antibiotic groups and substances on 79 ICUs participating in SARI project between 2006 and 2020, Germany [2/2].

| **Parameter** | **2006** | **2007** | **2008** | **2009** | **2010** | **2011** | **2012** | **2013** | **2014** | **2015** | **2016** | **2017** | **2018** | **2019** | **2020** | **Increase**  **2020 vs. 2006** | **% Increase**  **2020 vs. 2006** | **ALT per month**  **(95%CI)** | **p-value** | **ALT per year**  **(95%CI)** | **p-value** |
| --- | --- | --- | --- | --- | --- | --- | --- | --- | --- | --- | --- | --- | --- | --- | --- | --- | --- | --- | --- | --- | --- |
| No. ICU (N=79) | 44 | 43 | 43 | 45 | 43 | 43 | 52 | 50 | 46 | 46 | 49 | 45 | 44 | 43 | 38 |  |  |  |  |  |  |
| Antibiotic use density (DDD/100 patient days) (ATC-code) |  |  |  |  |  |  |  |  |  |  |  |  |  |  |  |  |  |  |  |  |  |
| Sulfonamides + Trimethoprime (J01E) | 1.7 | 2.0 | 2.7 | 2.6 | 2.3 | 2.1 | 3.5 | 3.4 | 4.2 | 4.6 | 4.3 | 3.8 | 3.4 | 3.3 | 3.3 | 1.6 | 96.2 | 0.013  (0.005, 0.019) | 0.001 | 0.145  (0.062, 0.229) | 0.001 |
| Tetracyclines (J01AA) | 1.4 | 2.4 | 2.3 | 2.9 | 3.6 | 2.6 | 2.7 | 2.6 | 2.3 | 3.2 | 3.3 | 2.5 | 2.5 | 2.3 | 2.3 | 1.0 | 71.5 | 0.001  (-0.005, 0.007) | 0.753 | 0.009  (-0.062, 0.079) | 0.808 |
| Tigecyclin (p) (J01AA12) | 0.4 | 1.4 | 1.4 | 1.8 | 2.2 | 1.8 | 1.7 | 1.7 | 1.4 | 2.1 | 2.1 | 1.6 | 1.5 | 1.6 | 1.6 | 1.2 | 327.0 | 0.002  (-0.002,0.007) | 0.341 | 0.027  (-0.031, 0.085) | 0.360 |
| Macrolides (J01FA) | 7.4 | 8.3 | 8.9 | 10.9 | 10.8 | 11.2 | 10.5 | 10.8 | 9.7 | 11.2 | 9.0 | 9.0 | 8.8 | 8.3 | 9.2 | 1.8 | 24.6 | -0.007  (-0.024, 0.011) | 0.446 | -0.090  (-0.299, 0.118) | 0.396 |
| Lincosamines (J01FF) | 2.5 | 2.1 | 2.3 | 2.7 | 2.3 | 2.8 | 2.3 | 2.3 | 2.3 | 2.9 | 2.0 | 1.4 | 1.2 | 1.7 | 1.3 | -1.2 | -48.6 | -0.006  (-0.009, -0.003) | <0.001 | -0.0698  (-0.107, -0.033) | <0.001 |
| Aminoglycosides (J01G) | 3.0 | 2.8 | 2.5 | 2.8 | 3.1 | 2.9 | 2.9 | 2.6 | 2.7 | 3.1 | 3.5 | 3.0 | 3.6 | 3.6 | 4.0 | 1.1 | 36.0 | 0.006  (-0.003, 0.015) | 0.221 | 0.066  (-0.045, 0.176) | 0.244 |
| Imidazolderivates(J01XD) | 6.6 | 6.0 | 5.3 | 4.9 | 4.6 | 4.7 | 4.7 | 4.4 | 4.1 | 4.8 | 3.9 | 3.6 | 3.2 | 2.9 | 2.5 | -4.1 | -62.3 | -0.025  (-0.036, -0.013) | <0.001 | -0.315  (-0.458, -0.173) | <0.001 |
| Tuberkulostatics (J01A) | 0.5 | 0.7 | 1.5 | 1.4 | 1.1 | 1.6 | 1.4 | 1.8 | 2.3 | 2.6 | 2.5 | 1.6 | 2.5 | 2.1 | 2.2 | 1.7 | 346.3 | 0.009  (0.003, 0.016) | 0.006 | 0.107  (0.031, 0.183) | 0.006 |
| Other antibiotics (J01XX) | 4.0 | 3.8 | 4.7 | 5.4 | 6.2 | 6.2 | 7.4 | 7.4 | 5.8 | 6.8 | 7.8 | 7.2 | 8.4 | 9.2 | 10.4 | 6.4 | 161.0 | 0.028  (0.016, 0.040) | <0.001 | 0.322  (0.177, 0.466) | <0.001 |
| Linezolid (p) (J01XX08) | 2.7 | 2.4 | 2.9 | 3.6 | 3.4 | 4.4 | 4.1 | 3.9 | 3.1 | 3.7 | 4.4 | 4.0 | 4.6 | 5.0 | 5.3 | 2.5 | 93.2 | 0.011 (0.004, 0.018) | 0.001 | 0.123 (0.051, 0.209) | 0.001 |
| Linezolid (o) (J01XX08) | 0.4 | 0.3 | 0.4 | 0.2 | 0.2 | 0.1 | 0.1 | 0.3 | 0.2 | 0.2 | 0.2 | 0.2 | 0.2 | 0.1 | 0.2 | -0.2 | -49.4 | -0.001 (-0.002, 0.0002) | 0.104 | -0.009 (-0.021, 0.003) | 0.131 |
| Daptomycin (p) (J01XX09) | 0.0 | 0.5 | 0.4 | 0.5 | 1.0 | 0.9 | 1.9 | 1.7 | 1.5 | 1.6 | 2.1 | 1.5 | 2.2 | 2.2 | 2.7 | 2.7 | 36120.5 | 0.013 (0.007, 0.020) | <.001 | 0.157 (0.080, 0.233) | <.001 |

No., number; ICU, intensive care unit; ; ATC-code, Anatomical Therapeutic Chemical (ATC) code; DDD, defined daily doses; p, parenteral; o, oral; ALT, Adjusted Linear Trend; CI, confidence interval; ND, not defined; *ALT per month/year, were calculated by GEE model with ICU-based data considering the cluster effect of the ICU.

**Supplemental Table 3**: Multivariate analyses for the outcome incidence density (ID) of Vancomycin-resistant *Enterococcus* *faecium* and *faecalis.* Model adjusted by time and Tetracyclines*/Other antibacterials**.

| Parameter | Category | VR*E. faecium* |  | VR*E. faecalis* |  |
| --- | --- | --- | --- | --- | --- |
|  |  | IRR (95%CI) | p-value | IRR (95%CI) | p-value |
| Model adjusted by time and Tetracyclines*/Other antibacterials** | | | | | |
| Time trend (linear) | per month | 1.015 (1.013-1.017) | <.0001 | 1.003 (0.998-1.008) | 0.247 |
| Tetracyclines (J01AA)* | per 1DDD/100 pd | 1.007 (1.003-1.011) | <.0001 | 0.997 (0.949-1.047) | 0.890 |
| Other antibacterials (J01XX)** | per 1DDD/100 pd | 1.009 (1.002-1.016) | 0.010 | 1.006 (0.959-1.055) | 0.808 |
| Carbapeneme (J01DH) | per 1DDD/100 pd | 1.007 (1.004-1.011) | <.0001 | - | - |
| Glycopeptide (J01XA) | per 1DDD/100 pd | 1.008 (1.002-1.014) | 0.010 | - | - |
| Imidazole derivatives (J01XD) | per 1DDD/100 pd | 1.026 (1.01-1.043) | 0.001 |  |  |
| Beta-lactamase sensitive penicillin (J01CE) | per 1DDD/100 pd | - | - | 0.879 (0.809-0.955) | 0.002 |

*Tigecycline (J01AA12) (69% of use in the group J01AA)

**Linezolid (J01XX08) & Daptomycin (J01XX09) (79% of use in the group J01XX in the year 2020)

**Supplemental Table 4**: Multivariate analyses for the outcome resistance rate of Vancomycin-resistant *Enterococcus* *faecium* and *faecalis.* Model adjusted by time and Tetracyclines*/Other antibacterials**.

| Parameter | Category | VR*E. faecium* |  | VR*E. faecalis* |  |
| --- | --- | --- | --- | --- | --- |
|  |  | IRR (95%CI) | p-value | IRR (95%CI) | p-value |
| Model adjusted by time and Tetracyclines*/Other antibacterials** | | | | | |
| Time trend (linear) | per month | 1.017 (1.014-1.019) | <.0001 | 1.003 (0.998-1.009) | 0.228 |
| Tetracyclines (J01AA)* | per 1DDD/100 pd | 1.002 (0.998-1.006) | 0.297 | 0.995 (0.946-1.047) | 0.855 |
| Other antibacterials (J01XX)** | per 1DDD/100 pd | 1.004 (0.995-1.012) | 0.415 | 1.003 (0.946-1.064) | 0.913 |
| Glycopeptide (J01XA) | per 1DDD/100 pd | 1.015 (1.003-1.027) | 0.014 | - | - |
| Aminoglycosides (J01G) | per 1DDD/100 pd | 1.013 (1.003-1.023) | 0.008 | - | - |
| Beta-lactamase sensitive penicillin (J01CE) | per 1DDD/100 pd | - | - | 0.915 (0.868-0.965) | 0.001 |

*Tigecycline (J01AA12) (69% of use in the group J01AA)

**Linezolid (J01XX08) & Daptomycin (J01XX09) (79% of use in the group J01XX in the year 2020)

**Supplemental Table 5**: Spearman correlation between antibiotic use of antibiotic groups [1/2].

| **Parameter (ATC-code)** |  | **t** | **J01CE** | **J01CA** | **J01CF** | **J01CR** | **J01DB** | **J01DC** | **J01DD** | **J01DE** | **J01DH** | **J01XA** | **J01MA** | **J01E** | **J01AA** | **J01FA** | **J01FF** | **J01G** | **J01XD** | **J01A** | **J01XX** |
| --- | --- | --- | --- | --- | --- | --- | --- | --- | --- | --- | --- | --- | --- | --- | --- | --- | --- | --- | --- | --- | --- |
| time | CC | 1.00 | 0.04 | -0.18 | 0.26 | 0.44 | 0.11 | -0.24 | -0.16 | 0.08 | 0.26 | 0.07 | -0.19 | 0.11 | 0.03 | -0.01 | -0.09 | -0.01 | -0.25 | 0.17 | 0.10 |
|  | p |  | 0.00 | <.01 | <.01 | <.01 | <.01 | <.01 | <.01 | <.01 | <.01 | <.01 | <.01 | <.01 | 0.01 | 0.42 | <.01 | 0.29 | <.01 | <.01 | <.01 |
| Beta-lactamase sensitive penicilline (J01CE) | CC | 0.04 | 1.00 | 0.05 | 0.11 | 0.02 | 0.09 | 0.02 | -0.01 | 0.00 | 0.04 | 0.02 | -0.02 | 0.08 | 0.00 | 0.01 | 0.09 | 0.06 | -0.01 | 0.06 | 0.07 |
|  | p | 0.00 |  | <.01 | <.01 | 0.03 | <.01 | 0.08 | 0.60 | 0.96 | 0.00 | 0.17 | 0.05 | <.01 | 0.74 | 0.29 | <.01 | <.01 | 0.26 | <.01 | <.01 |
| Penicillins with extended spectrum (J01CA) | CC | -0.18 | 0.05 | 1.00 | 0.03 | -0.16 | 0.10 | 0.01 | 0.10 | 0.01 | 0.01 | 0.04 | 0.00 | 0.02 | 0.02 | 0.03 | 0.05 | 0.08 | 0.06 | 0.03 | 0.00 |
|  | p | <.01 | <.01 |  | 0.01 | <.01 | <.01 | 0.24 | <.01 | 0.29 | 0.45 | 0.00 | 0.97 | 0.05 | 0.06 | 0.00 | <.01 | <.01 | <.01 | 0.01 | 0.88 |
| Beta-lactamase resistant penicilline (J01CF) | CC | 0.26 | 0.11 | 0.03 | 1.00 | 0.10 | 0.06 | -0.03 | -0.06 | 0.07 | 0.06 | 0.09 | -0.05 | 0.07 | -0.06 | -0.04 | 0.01 | 0.08 | -0.18 | 0.11 | 0.13 |
|  | p | <.01 | <.01 | 0.01 |  | <.01 | <.01 | 0.01 | <.01 | <.01 | <.01 | <.01 | <.01 | <.01 | <.01 | 0.00 | 0.28 | <.01 | <.01 | <.01 | <.01 |
| Combinations of penicillins, incl. beta-lactamase inhibitors (J01CR) | CC | 0.44 | 0.02 | -0.16 | 0.10 | 1.00 | 0.01 | -0.15 | 0.01 | 0.08 | 0.13 | 0.04 | 0.05 | 0.06 | 0.06 | 0.21 | 0.05 | -0.01 | -0.07 | 0.04 | 0.03 |
|  | p | <.01 | 0.03 | <.01 | <.01 |  | 0.34 | <.01 | 0.58 | <.01 | <.01 | 0.00 | <.01 | <.01 | <.01 | <.01 | <.01 | 0.65 | <.01 | 0.00 | 0.01 |
| First-generation cephalosporins (J01DB) | CC | 0.11 | 0.09 | 0.10 | 0.06 | 0.01 | 1.00 | -0.11 | -0.06 | -0.05 | -0.05 | -0.10 | -0.12 | -0.02 | -0.02 | -0.06 | -0.01 | -0.03 | 0.02 | 0.00 | 0.06 |
|  | p | <.01 | <.01 | <.01 | <.01 | 0.34 |  | <.01 | <.01 | <.01 | <.01 | <.01 | <.01 | 0.06 | 0.06 | <.01 | 0.47 | 0.00 | 0.07 | 0.98 | <.01 |
| Second-generation cephalosporins (J01DC) | CC | -0.24 | 0.02 | 0.01 | -0.03 | -0.15 | -0.11 | 1.00 | -0.04 | -0.01 | -0.15 | -0.10 | 0.03 | -0.13 | -0.01 | -0.11 | 0.08 | -0.05 | 0.20 | -0.01 | -0.05 |
|  | p | <.01 | 0.08 | 0.24 | 0.01 | <.01 | <.01 |  | 0.00 | 0.23 | <.01 | <.01 | 0.01 | <.01 | 0.54 | <.01 | <.01 | <.01 | <.01 | 0.44 | <.01 |
| Third-generation cephalosporins (J01DD) | CC | -0.16 | -0.01 | 0.10 | -0.06 | 0.01 | -0.06 | -0.04 | 1.00 | 0.02 | 0.09 | 0.15 | 0.20 | 0.09 | 0.09 | 0.18 | 0.16 | 0.09 | 0.22 | 0.01 | 0.03 |
|  | p | <.01 | 0.60 | <.01 | <.01 | 0.58 | <.01 | 0.00 |  | 0.03 | <.01 | <.01 | <.01 | <.01 | <.01 | <.01 | <.01 | <.01 | <.01 | 0.48 | 0.01 |
| Fourth-generation cephalosporins (J01DE) | CC | 0.08 | 0.00 | 0.01 | 0.07 | 0.08 | -0.05 | -0.01 | 0.02 | 1.00 | 0.15 | 0.17 | 0.09 | 0.05 | 0.05 | -0.01 | 0.04 | 0.16 | -0.03 | 0.14 | 0.05 |
|  | p | <.01 | 0.96 | 0.29 | <.01 | <.01 | <.01 | 0.23 | 0.03 |  | <.01 | <.01 | <.01 | <.01 | <.01 | 0.26 | 0.00 | <.01 | 0.01 | <.01 | <.01 |
| Carbapenemes (J01DH) | CC | 0.26 | 0.04 | 0.01 | 0.06 | 0.13 | -0.05 | -0.15 | 0.09 | 0.15 | 1.00 | 0.44 | 0.15 | 0.21 | 0.18 | 0.09 | -0.02 | 0.21 | 0.03 | 0.43 | 0.16 |
|  | p | <.01 | 0.00 | 0.45 | <.01 | <.01 | <.01 | <.01 | <.01 | <.01 |  | <.01 | <.01 | <.01 | <.01 | <.01 | 0.13 | <.01 | 0.01 | <.01 | <.01 |
| Glycopeptides (J01XA) | CC | 0.07 | 0.02 | 0.04 | 0.09 | 0.04 | -0.10 | -0.10 | 0.15 | 0.17 | 0.44 | 1.00 | 0.16 | 0.19 | 0.11 | -0.01 | 0.09 | 0.32 | 0.02 | 0.18 | 0.19 |
|  | p | <.01 | 0.17 | 0.00 | <.01 | 0.00 | <.01 | <.01 | <.01 | <.01 | <.01 |  | <.01 | <.01 | <.01 | 0.44 | <.01 | <.01 | 0.05 | <.01 | <.01 |
| Fluorochinolone (J01MA) | CC | -0.19 | -0.02 | 0.00 | -0.05 | 0.05 | -0.12 | 0.03 | 0.20 | 0.09 | 0.15 | 0.16 | 1.00 | 0.06 | 0.12 | 0.12 | 0.15 | 0.13 | 0.10 | 0.14 | 0.07 |
|  | p | <.01 | 0.05 | 0.97 | <.01 | <.01 | <.01 | 0.01 | <.01 | <.01 | <.01 | <.01 |  | <.01 | <.01 | <.01 | <.01 | <.01 | <.01 | <.01 | <.01 |
| Sulfonamides + Trimethoprime (J01E) | CC | 0.11 | 0.08 | 0.02 | 0.07 | 0.06 | -0.02 | -0.13 | 0.09 | 0.05 | 0.21 | 0.19 | 0.06 | 1.00 | 0.11 | 0.08 | 0.08 | 0.15 | -0.06 | 0.13 | 0.10 |
|  | p | <.01 | <.01 | 0.05 | <.01 | <.01 | 0.06 | <.01 | <.01 | <.01 | <.01 | <.01 | <.01 |  | <.01 | <.01 | <.01 | <.01 | <.01 | <.01 | <.01 |

Correlation coefficients were calculated based of monthly ICU-based aggregated data; ATC-code, Anatomical Therapeutic Chemical (ATC) code; CC, Spearman correlation coefficient; p, p-value for CC;

**Supplemental Table 5**: Spearman correlation between antibiotic use of antibiotic groups [2/2].

| **Parameter (ATC-code)** |  | **t** | **J01CE** | **J01CA** | **J01CF** | **J01CR** | **J01DB** | **J01DC** | **J01DD** | **J01DE** | **J01DH** | **J01XA** | **J01MA** | **J01E** | **J01AA** | **J01FA** | **J01FF** | **J01G** | **J01XD** | **J01A** | **J01XX** |
| --- | --- | --- | --- | --- | --- | --- | --- | --- | --- | --- | --- | --- | --- | --- | --- | --- | --- | --- | --- | --- | --- |
| Tetracyclines (J01AA) | CC | 0.03 | 0.00 | 0.02 | -0.06 | 0.06 | -0.02 | -0.01 | 0.09 | 0.05 | 0.18 | 0.11 | 0.12 | 0.11 | 1.00 | 0.08 | 0.05 | 0.08 | 0.07 | 0.17 | 0.04 |
|  | p | 0.01 | 0.74 | 0.06 | <.01 | <.01 | 0.06 | 0.54 | <.01 | <.01 | <.01 | <.01 | <.01 | <.01 |  | <.01 | <.01 | <.01 | <.01 | <.01 | 0.00 |
| Macrolides (J01FA) | CC | -0.01 | 0.01 | 0.03 | -0.04 | 0.21 | -0.06 | -0.11 | 0.18 | -0.01 | 0.09 | -0.01 | 0.12 | 0.08 | 0.08 | 1.00 | 0.07 | 0.02 | 0.13 | -0.02 | -0.03 |
|  | p | 0.42 | 0.29 | 0.00 | 0.00 | <.01 | <.01 | <.01 | <.01 | 0.26 | <.01 | 0.44 | <.01 | <.01 | <.01 |  | <.01 | 0.03 | <.01 | 0.06 | 0.01 |
| Lincosamines (J01FF) | CC | -0.09 | 0.09 | 0.05 | 0.01 | 0.05 | -0.01 | 0.08 | 0.16 | 0.04 | -0.02 | 0.09 | 0.15 | 0.08 | 0.05 | 0.07 | 1.00 | 0.11 | 0.03 | 0.00 | 0.08 |
|  | p | <.01 | <.01 | <.01 | 0.28 | <.01 | 0.47 | <.01 | <.01 | 0.00 | 0.13 | <.01 | <.01 | <.01 | <.01 | <.01 |  | <.01 | 0.02 | 0.99 | <.01 |
| Aminoglycosides (J01G) | CC | -0.01 | 0.06 | 0.08 | 0.08 | -0.01 | -0.03 | -0.05 | 0.09 | 0.16 | 0.21 | 0.32 | 0.13 | 0.15 | 0.08 | 0.02 | 0.11 | 1.00 | -0.02 | **0.20** | 0.16 |
|  | p | 0.29 | <.01 | <.01 | <.01 | 0.65 | 0.00 | <.01 | <.01 | <.01 | <.01 | <.01 | <.01 | <.01 | <.01 | 0.03 | <.01 |  | 0.03 | **<.01** | <.01 |
| Imidazolderivates(J01XD) | CC | -0.25 | -0.01 | 0.06 | -0.18 | -0.07 | 0.02 | 0.20 | 0.22 | -0.03 | 0.03 | 0.02 | 0.10 | -0.06 | 0.07 | 0.13 | 0.03 | -0.02 | 1.00 | -0.02 | -0.10 |
|  | p | <.01 | 0.26 | <.01 | <.01 | <.01 | 0.07 | <.01 | <.01 | 0.01 | 0.01 | 0.05 | <.01 | <.01 | <.01 | <.01 | 0.02 | 0.03 |  | 0.08 | <.01 |
| Tuberkulostatics (J01A) | CC | 0.17 | 0.06 | 0.03 | 0.11 | 0.04 | 0.00 | -0.01 | 0.01 | 0.14 | 0.43 | 0.18 | 0.14 | 0.13 | 0.17 | -0.02 | 0.00 | 0.20 | -0.02 | 1.00 | 0.14 |
|  | p | <.01 | <.01 | 0.01 | <.01 | 0.00 | 0.98 | 0.44 | 0.48 | <.01 | <.01 | <.01 | <.01 | <.01 | <.01 | 0.06 | 0.99 | <.01 | 0.08 |  | <.01 |
| Other antibiotics (J01XX) | CC | 0.10 | 0.07 | 0.00 | 0.13 | 0.03 | 0.06 | -0.05 | 0.03 | 0.05 | 0.16 | 0.19 | 0.07 | 0.10 | 0.04 | -0.03 | 0.08 | 0.16 | -0.10 | 0.14 | 1.00 |
|  | p | <.01 | <.01 | 0.88 | <.01 | 0.01 | <.01 | <.01 | 0.01 | <.01 | <.01 | <.01 | <.01 | <.01 | 0.00 | 0.01 | <.01 | <.01 | <.01 | <.01 |  |

Correlation coefficients were calculated based of monthly ICU-based aggregated data; ATC-code, Anatomical Therapeutic Chemical (ATC) code; CC, Spearman correlation coefficient; p, p-value for CC;
